# Spanish Version of the Family Health Behavior Scale: Adaptation and Validation

**DOI:** 10.3390/ijerph16050810

**Published:** 2019-03-06

**Authors:** María-Dolores Lanzarote-Fernández, José-Francisco Lozano-Oyola, Montserrat Gómez-de-Terreros-Guardiola, Isabel Avilés-Carvajal, Rafael J. Martínez-Cervantes, Jennette Palcic Moreno

**Affiliations:** 1 School of Psychology, University of Seville, C/Camilo José Cela s/n, 41018 Seville, Spain; flozano@us.es (J.-F.L.-O.); guardi@us.es (M.G.-d.-T.-G.); isabelaviles@us.es (I.A.-C.); rmcervan@us.es (R.J.M.-C.); 2 Baylor College of Medicine, 6655 Travis Mailstop, Houston, TX 77030, USA; palcic@bcm.edu

**Keywords:** family, health behavior, mealtime routines, paediatric obesity, physical activity, scale

## Abstract

Different studies around the world indicate that the percentages of overweight and obesity in childhood and adolescence are high. In this context, it would be useful to have a common, valid, and reliable instrument to assess health behaviors of families that allows comparisons of data from different countries. The objective is the adaptation of a Spanish version of the Family Health Behavior Scale (FHBS). The questionnaire originally developed by Moreno group was translated and adapted following the International Test Commission protocol. Its psychometric properties were evaluated through analysis of internal consistency, factor analysis and other evidences of validity. The Spanish version of the FHBS demonstrated adequate reliability coefficients, and its factor structure sufficiently replicated that obtained by the original measurement. The results suggested that the adapted version of the questionnaire was an adequate and valid measure for the evaluation of family health behaviors related to the prevention of overweight and obesity.

## 1. Background

Overweight and obesity rates in children and adolescents are currently a concern both in developed countries and in those with emerging economies [1]; due to the complications and associated risks they may pose, as well as the impact on physical [1,2] and psychological [3,4,5] quality of life of these future adults. Similar warnings have been raised in Spain, where different studies indicated that the percentages of overweight and obesity in child and adolescent populations have stabilized at high levels [6,7] and are increasing at lower ages and at lower economic levels [8].

Assuming overweight and obesity are largely preventable [9], the final report of the *Commission on Ending Childhood Obesity* stated, “Academic institutions can contribute to addressing childhood obesity through studies on biological, behavioural, and environmental risk factors and determinants, and the effectiveness of interventions in each of these.” ([10], p. 37). To prevent instead of acting once overweight and obesity have appeared, families’ healthy habits have an important role to play. In this sense, a significant relationship has been found between the intergenerational transmission of obesity and family lifestyles that cannot be explained exclusively by genetic components [11,12]. Therefore, early detection and intervention on unhealthy family behaviors would help to reduce the prevalence of weight problems.

Health professionals have been concerned with the assessment and measurement of behavioral factors and habits related to overweight in childhood and adolescence. Between the several instruments of the parents’ mealtime routines that Vaughn, Tabak, Bryant, and Ward [13] have revised, the Family Health Behavior Scale (FHBS) developed by Moreno et al. [14] allows an efficient assessment of family behaviors related to obesity in childhood during primary and secondary education years. It contains 27 items and provides information on four areas: Parent Behaviors, Physical Activity, Child Behaviors, and Mealtime Routines. The questionnaire evidenced adequate validity and reliability data for USA population.

From an international perspective, the problem with the evaluation and measurement of behavioral and social factors is that they are strongly conditioned by cultural and linguistic determinants, unlike physical and biological measures. This makes the comparative use between countries of behavioral scales difficult. The existence of a common, brief, and valid instrument to assess family health behaviors would be useful to understand the possible role of countries socio-cultural factors on children and adolescents’ overweight and obesity. 

Instruments as the FHBS could play that role, but there is no similar questionnaire in Spanish that briefly and effectively measures family behavioral habits related to unhealthy children’s weights. In order to perform this assessment of family health behaviors in Spain with a reliable and valid instrument, we consider the adaptation and validation of the FHBS to the Spanish language and culture as the objective of this study. 

## 2. Methods

### 2.1. Participants

The sample recruitment was tailored to the characteristics of the original sample used by Moreno et al. [14] in the development and validation of the original FHBS scale in the USA. Our sample was composed of 360 caregivers of children of school age ranging from 5 to 12 years (*M* = 8.4, *SD* = 2.5), of which 44.4% were girls.

Caregivers provided the age, sex, height, and weight of the children. The children’s body mass indexes (BMI) adjusted for the sex and age were calculated using the application developed by Romero [15]. The median of the BMI percentile of the minors was 59.1 (*SD* = 31.6). According to these percentiles, 64.3% of the children showed a healthy weight, 13.0% were overweight, 13.8% were obese, and 8.9% were underweight. No statistical differences were found between the distributions of BMI groups in both samples (*K-S* Test D = 0.5 *p* = 0.699). Table 1 compares the data of the sample of children participating in this study with those of the original sample [14].

### 2.2. Measures

Parents or guardians communicated data on their children and adolescents’ age, sex, height, and weight. The family health habits were measured with the FHBS questionnaire developed by Moreno et al. [14] that evaluates the healthy behaviors of the family related to food and physical exercise. The scale has 27 items divided into four subscales, one referring to Parent Behaviors (10 items), and the remaining three referred to Physical Activity (6 items), Mealtime Routines (5 items), and Children Behaviors (6 items). Each item is answered from a five-point Likert scale that goes from 0 (almost never) to 4 (nearly always). The total scores per subscale are obtained by adding the item scores, after reverse scoring the negative items, so that a higher score means a higher frequency of healthy behaviors.

### 2.3. Procedure 

For the process of adaptation and validation of the FHBS questionnaire to Spanish, the criteria proposed by the International Test Commission [16] and Hambleton [17] were followed. Linguistic equivalence was sought through a process of translation and retro-translation. Cultural equivalence was assessed through interviews and consultations with experts. Finally, the statistical equivalence was verified analysing the psychometric properties of the questionnaire administered to a Spanish sample with similar characteristics to those of the original study in the USA.

#### 2.3.1. Adaptation

Once the agreement of the original authors was obtained, a professional translated the items of the FHBS from English to Spanish. After comparing the Spanish translation with the original version of the questionnaire, a pilot version was obtained. This pilot version of the questionnaire was administered in individual interviews with parents of children enrolled in primary education. Parents were asked to review the items, identify any words or concepts in the questionnaire that they did not understand. After these contributions, slight modifications were made. In parallel, experts in pediatrics and nutrition from a third level hospital were consulted to evaluate the content of the items of the translated questionnaire. The most relevant change that occurred with respect to the original questionnaire was that the item “My child eats three meals a day” was modified to “My child eats four or five meals a day”, adjusting it to Spanish cultural habits. Once the final version of the questionnaire was prepared, it was sent to another translator to carry out a retro-translation from Spanish to English. With the exception of the aforementioned item, it was found that both the literal meaning and the sense of the items of the Spanish version were coincident with the original. In this way, the linguistic and semantic equivalence of the Spanish version of the FHBS questionnaire with respect to the original was assumed, and it was considered ready to be administered.

#### 2.3.2. Validation

Caregivers of children enrolled in primary schools of the province of Seville, Southern Spain, were recruited. All the schools were from urban, middle-class populations located in the metropolitan area of Seville (688.171 inhabitants). Most of them contacted through a face-to-face procedure, in which undergraduate students of the University of Seville went to the participating schools. Only a small sample (*n* = 27) were contacted through the Internet once the school principals authorized the survey, without apparent impact on the resulting data. In all cases, together with the distribution of the FHBS questionnaires, the parents or guardians of the minors provided informed consent to participate. The Ethics Committee of the institution where researchers collaborate authorized the study (Virgen del Rocio Hospital, Seville). 

#### 2.3.3. Data Analysis

The analysis techniques used were: (a) descriptive analysis of the items; (b) analysis of the internal consistency of the subscales and the total scale of the FHBS using Cronbach’s alpha coefficients; (c) factor analysis through the FACTOR program (Ver. 10.3.01 XP [18,19]); (d) calculation of factor scores, reliability estimates and their correlations and with the BMI measures; and (e) binomial logistic regression analysis to predict from the factor scores the dichotomized classification between the healthy weight group against overweight and obese groups.

## 3. Results

### 3.1. Descriptive Analysis 

Most of the items presented mean values above 2 in the items’ scale from 0 to 4, except in the case of item number 5 (“My child eats frequently during the day”, from the Children Behaviors scale) whose 95% confidence interval around their mean did not exceed the threshold. This is consistent with the analysis of corrected item-total correlations, which only in item 5 showed a clearly inadequate level (*r* = 0.053). In all the items the maximum range of responses was found, with 95.8% of the sample (345 subjects) answering all the items and only 24 blank responses appeared, distributed in 16 of the items. As for the skewness and kurtosis of the responses to the items, it was found that in 17 of the items the absolute value of 1.00 was exceeded in the asymmetry indexes, in the kurtosis indices or in both, indicating that their distributions do not correspond to those of a normal curve. The multivariate analysis of Mardia [20], corroborated statistically the absence of normality in the case of kurtosis (*B* = 34.21, *p* < 0.0001). 

After inverting the scores of the negative items, new scores were obtained for each subscale, following the original item distribution of Moreno et al. [14], as well as for the total of the FHBS. To facilitate the interpretation of these scores, they were linearly transformed to scales from 0 to 100. The descriptive results of these scores show averages between 59.08 and 70.65, except for the Subscale of Mealtime Routines that rises to 91.98. This subscale is also the one with the lowest standard deviation (*SD* = 10.14). Regarding the distributions of these scores, in all cases, the skewness and kurtosis indexes are lower than an absolute value of 1.00, except for the Mealtime Routines subscale, which has a clearly skewed distribution towards the higher values.

### 3.2. Internal Consistency

Cronbach’s alpha coefficients showed sufficient internal consistency for both the FHBS Total scale (α = 0.746) and the Parent Behaviors subscale (α = 0.761). In the rest of the subscales, the value of 0.70 was not reached, being especially low the value of the subscale of Mealtime Routines (Table 2).

### 3.3. Factor Analysis

Given the behavior in terms of skewness and kurtosis of the items, a factor analysis was made from the polychoric correlation matrix, as recommended by Lorenzo-Seva and Ferrando [18,19]. This analysis was limited to four factors to assess the validity of the model obtained by Moreno et al. [1], using the procedure of robust weighted squares (Unweighted Least Squares, ULS) and a method of oblique rotation Promin [21] to achieve greater factorial simplicity. As a final criterion, the solution was limited to a maximum of 100 iterations or to a convergence value of 0.00001.

The analysis of the adequacy of the correlation matrix was considered satisfactory, according to the Bartlett sphericity test (*M* = 0.00134, χ^2^ = 2208.5, *df* = 351; *p* = 0.00001), and the Kaiser–Meyer–Olkin index (*KMO* = 0.70537). This result justified the limitation imposed on the four-factor solution, which explained up to 46.3% of the variance found in the 27-item questionnaire. The adjustment of the four-factor model was satisfactory (*GFI* = 0.96; *RMSR* = 0.06), showing an adequate Bentler’s simplicity index (*S* = 0.98).

As can be seen in the rotated factor matrix of factorial loadings (Table 3), the content of the four factors extracted coincided with those of the original work: Children Behaviors, Physical Activity, Mealtime Routines, and Parent Behaviors. Considering only the items with absolute loading greater than 0.40, following the same criteria as Moreno et al. [14], the classification of the items coincided in 81.5% of cases with the original (22 of 27 items). Of the five items whose results do not coincide with the original solution, in four of them it is due to their factor loadings being lower than 0.40 (items 3, 5, 24, and 27), while one item loaded on the Mealtime Routines factor rather than the Parent Behaviors factor (item 4: “My child has help choosing healthy foods”).

Unlike the factorial solution found by Moreno et al. [14] the Child Behaviors factor explained the highest proportion of variance (24.0%), while the Parent Behaviors factor explained the least amount of variance (8.2%). Reliability estimators based on factor analysis (MacDonald’s Omega, Ω) show all satisfactory values for the four factors, and higher than 0.79 in all of the factor scores.

The analysis of the correlations (Table 4) between the scores of the different factors showed that the Child Behaviors factor was not related with the other three factors (Parent Behaviors, Mealtime Routines, and Physical Activity), which were related with each other. The correlations of the scores on the four factors of FHBS with the BMI were negative and significant for the subscales of Parent Behaviors, Physical Activity, and Children Behaviors, which shows that lower scores on healthy family habits are associated with a slight increase in BMI.

### 3.4. Factor Scores

**Figure 1:**
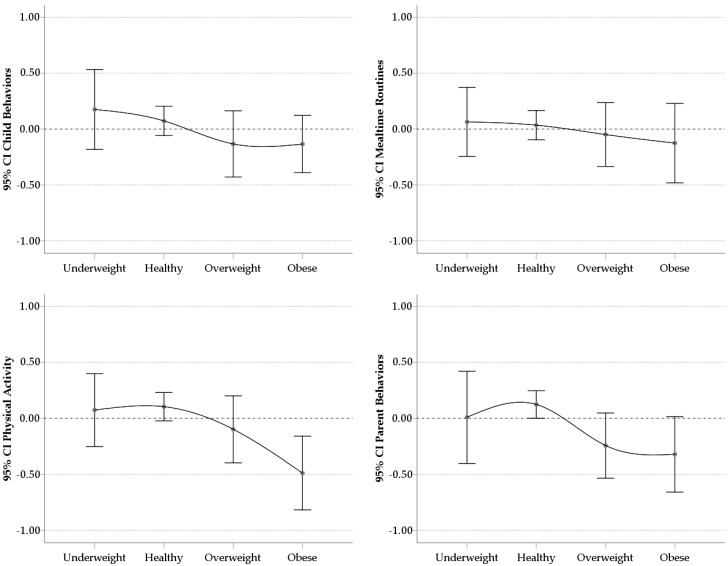
Means and 95% Confidence Intervals of the factor scores for the BMI classification groups.

The analysis of the means and 95% confidence intervals of the factor scores in the four factors according to the different groups of classification of the BMI (underweight, healthy weight, overweight, and obese), is shown in Figure 1. These scores were typified with an average around 0.00 and a standard deviation of 1.00. The scores of the groups of children with overweight and obesity were below the average (negative), while the groups of healthy and underweight were above the average (positive), being in all cases the lowest scores for the obese group, indicating more obesogenic family health habits. Figure 1 shows a curved descending pattern from the healthy weight group to the obese group in the family health habits. The underweight group scores similar to the healthy weight group. In any case, the distribution of the means of the scores of Child Behaviors and Mealtime Routines was overlapping between the different groups taking into account the 95% confidence intervals around the means showing no discriminative power. However, in the case of the scores of the Physical Activity and Parent Behaviors, the healthy weight group showed an average outside the confidence interval of the obese group.

### 3.5. Validity

With the aim of replicating the analysis of the possible validity of measures of family health habits to predict overweight and obesity, a binomial logistic regression model was carried out following the procedures of Moreno et al. [14]. As in the original study, the children classified with underweight were excluded, resulting in a sample size of 316 for this analysis. As a criterion variable, we differentiated two groups: those classified as overweight and obese on one side, from those classified as healthy weight. Using this classification and the total scores in the FHBS scale as a predictor variable, the results indicated that each point of increase in the FHBS scale implied a decrease of 4.3% in the probability of being in the overweight or obese group (*OR* = 0.957, 95% *CI* 0.934–0.980; *p* < 0.001). In comparison, the original study demonstrated a 3.9% decrease in the likelihood of being overweight or obese for every point increase. This model correctly predicted the BMI classification of 71.5% of the participants, which was higher than the 62.8% found in the original study.

Bivariate correlation analyses were performed to determine if the *z*BMI was significantly related to the subscales and the total score of the FHBS. For these analyses, those classified as underweight or healthy weight were excluded, in order to replicate the original study [14]. This resulted in a subsample of 93 children with a BMI equal to or greater than the 85th percentile. The results indicated that in this group *z*BMI was only significantly and inversely related to the Physical Activity scale (*r* = −0.279; *p* = 0.008). The scales of Parent Behaviors, Children Behaviors, and Mealtime Routines, as well as the total scores in the FHBS, did not correlate significantly with *z*BMI (with values between −0.163 and 0.051). The analysis of the bivariate correlations between *z*BMI and the scores obtained from the factor analysis offered similar results.

## 4. Discussion

The Spanish adaptation of the Family Health Behavior Scale [14] proved to be adequate and useful as an instrument to detect behaviors related with the risk of overweight or obesity. It is a short scale to be used by any professional who works with children and wanted to assess their family health habits. 

The comparisons with the results of the original study of Moreno et al. [14] showed similarities and differences. The classification of the items obtained from the factor analysis reproduces sufficiently the content of the items of the original study, with four clearly differentiated areas: Child Behaviors, Parent Behaviors, Physical Activity, and Mealtime Routines. The results of the factor analysis show good indexes of adjustments and the factor scores offer adequate estimates of reliability, showing as valid alternatives to the direct scores in the FHBS. Regarding the values of internal consistency, these were lower than those of the original study, both in the total scores of the FHBS and in the scores of the different subscales. However, the greatest consistency was found in the measure of Parent Behaviors and Total Scores, as in the original study. Conversely, Mealtime Routines was the least consistent score in the Spanish sample evaluated in this study, showing that perhaps the items of this subscale are not discriminative for this population. In relation to the variance explained by each of the factors, the Child Behaviors measure were the most determinant in the Spanish population, compared to the Parent Behaviors that were the most important in the USA population. 

It stands out that the subscale of Mealtime Routines did not provide much information in this Spanish sample, since all the subjects demonstrated high scores, failing to discriminate between participants with more or less healthy habits. It may be due to differences associated to Mediterranean culture on meals schedule in Spain, determined by work and school timeframe that facilitate well-established family customs. Especially in southern Spain, it is usual to have shared mealtime routines: e.g., most of the families eat at the table and children do not choose their meals. 

On the other hand, the correlations analysis revealed significant relationships between the BMI measures and all factors except for Mealtime Routines. Taking into account the four groups considered according to their BMI (underweight, healthy weight, overweight, and obese), the results showed a consistent trend with the hypothesis that worse family health habits were more common among the children with overweight and obesity. In addition, the binomial logistic regression model reproduced similar results to those of the original study. Thus, the total scores in the FHBS significantly predicted the weight classification of the healthy weight group and the overweight and obese groups. When the analysis focused only on the group of children with overweight and obesity, as in the original study, Physical Activity was significantly and inversely related to measures of BMI, showing convergent validity. In contrast, the correlations between the zBMI scores with the total scores of the FHBS and those of the subscale of Parent Behaviors were not significant, unlike the original study. 

One limitation of this study would be that parents provided the anthropometric measures (height and weight), and because of that, they may be inaccurate. However, this scale can be considered a primary prevention tool because it can detect unhealthy behaviors before the increase in BMI. A follow-up assessment protocol included at different periods could detect the risk of overweight and obesity. An educational program that promotes healthy behaviors such as those included in this scale could keep a healthy weight. Obviously, an evaluation made exclusively with one scale of family behavioral habits is incomplete, so sociodemographic and personal variables must be taken into account. For future studies, it would be desirable to relate the FHBS scores with more precise anthropometric and sociodemographic measurements.

## 5. Conclusions

We present a valid and reliable instrument applied to a large sample of children with a wide range of age. It is a user-friendly scale to be answered by parents and guardians. It measures four categories of healthy behaviors with only 27 items in Spanish language. As in the original Moreno’s paper, the FHBS helps to discriminate between overweight and healthy weight children and correlates significantly with BMI.

The resulting scale should allow for international comparisons with minor language and cultural adaptations regarding family health habits related to obesity and overweight. This scale would be useful to detect and prevent obesity and overweight in any context, such as educational, social, or clinical.

## Figures and Tables

**Table 1:** Comparison of the present study and Moreno et al. [14] sample characteristics.

Title	Moreno et al.	Present Study
Sample size	310	360
Age Rank	5–12	5–12
Mean age (*SD*)	8.7 (2.3)	8.4 (2.5)
Percentage of girls	51.0%	44.4%
Mean of -BMI Percentile (*SD*)	63.6 (33.0)	59.1 (31.6)
Weight Classification According to BMI		
Underweight	7.3%	8.9%
Healthy weight	54.0%	64.3%
Overweight	18.3%	13.0%
Obese	20.3%	13.8%

**Table 2:** Comparison of internal consistency (α) in the present study and Moreno et al. [14].

FHBS Subscales	Moreno et al.	Present Study
Total	0.830	0.746
Parent Behaviors	0.850	0.761
Physical Activity	0.750	0.613
Mealtime Routines	0.770	0.454
Child Behaviors	0.740	0.682

**Table 3:** Matrix of rotated factorial loadings, factors’ eigenvalues, explained variances, and reliability estimates.

Items	Factors			
	I	II	III	IV
**I. Child Behaviors**				
17. My child is influenced to eat unhealthy foods by other kids ^a^	0.81			
14. My child sneaks food ^a^	0.79			
16. My child eats when he/she feels sad, mad, or nervous ^a^	0.74			
10. My child is offered unhealthy foods by other family members ^a^	0.54			
7. My child frequently asks for unhealthy snacks ^a^	0.52			
5. My child eats frequently throughout the day ^a^				
**II. Physical Activity**				
8. My child is physically active for at least 30 min daily		0.77		
2. My child participates in sports (swimming, football, gymnastics, dance, etc.)		0.71		
6. My child participates in physical activities with us		0.47		
11. My child plays outside		0.41		
3. My child prefers indoor activities over outdoor activities ^a^				
27. I participate in physical activity with my child ^b^				
**III. Mealtime Routines**				
13. My child stays seated at the table			0.81	
12. My child eats meals at a routine time			0.75	
9. My child eats meals at the table			0.69	
15. My child eats four or five meals a day			0.63	
1. My child eats breakfast daily			0.51	
4. My child is assisted with making healthy food choices ^b^			0.41	
**IV. Parent Behaviors**				
20. I eat low calorie, low fat foods				0.77
18. I make low calorie, low fat foods when cooking for my family				0.70
22. I choose low calorie healthy options at fast food or at restaurants				0.62
23. I eat vegetables				0.48
26. I teach my child about healthy food choices				0.48
21. I keep unhealthy food out of sight of my child				0.47
19. I offer my child a healthy alternative when he/she asks for junk food				0.46
25. I serve fresh fruits and vegetables				0.40
24. I work out, exercise, or participate in physical activity				
Eigenvalues	5.3	2.9	2.1	1.8
Percentage of Explained Variance	24.0	13.4	9.2	8.2
Factor reliability estimators (Ω)	0.83	0.79	0.84	0.85

^a^ Items with reversed scores. ^b^ Item of the original questionnaire with a different classification.

**Table 4:** Matrix of correlations between the scores obtained from the factor analysis and the BMI scores.

Factor Scores	I	II	III	IV
I—Child Behaviors				
II—Physical Activity	0.026			
III—Mealtime Routines	0.079	0.305 **		
IV—Parent Behaviors	0.089	0.379 **	0.518 **	
BMI	−0.106 *	−0.138 *	−0.073	−0.151 **

* *p* < 0.05; ** *p* < 0.01; *N* = 360.
